# Task Performance and Meta-Cognitive Outcomes When Using Activity Workstations and Traditional Desks

**DOI:** 10.3389/fpsyg.2016.00957

**Published:** 2016-06-21

**Authors:** June J. Pilcher, Victoria C. Baker

**Affiliations:** Department of Psychology, Clemson University, Clemson, SCUSA

**Keywords:** **:** sedentary lifestyle, physical activity, cognitive performance, affect, morale

## Abstract

The purpose of the current study is to compare the effects of light physical activity to sedentary behavior on cognitive task performance and meta-cognitive responses. Thirty-eight undergraduate students participated in the study. The participants used a stationary bicycle with a desk top and a traditional desk while completing two complex cognitive tasks and measures of affect, motivation, morale, and engagement. The participants pedaled the stationary bicycle at a slow pace (similar in exertion to a normal walking pace) while working. The results indicated that cognitive task performance did not change between the two workstations. However, positive affect, motivation, and morale improved when using the stationary bicycle. These results suggest that activity workstations could be implemented in the work place and in educational settings to help decrease sedentary behavior without negatively affecting performance. Furthermore, individuals could experience a positive emotional response when working on activity workstations which in turn could help encourage individuals to choose to be more physical active during daily activities.

## Introduction

It is well known that regular physical activity has positive health benefits such as improved cardiovascular functioning, decreased body weight, and a more positive outlook (Warburton et al., 2006). Regular moderate to vigorous physical activity alone; however, does not seem to be the answer to long-term good health. Even in people exercising regularly, the amount of sedentary behavior is related to chronic disease (Owen et al., 2009) and physical frailty (Song et al., 2015). As such, it is important to consider factors that may contribute to rates of physical activity (Hagströmer et al., 2014).

Most adults are generally inactive (Sisson and Katzmarzyk, 2008) with the amount of physical activity decreasing with age and with lack of understanding of the health benefits of exercising (Mullineaux et al., 2001). There is also increasing evidence that to better understand the effects of activity levels we must consider how activity is incorporated throughout the day (Tudor-Locke and Schuna, 2012). One example is in sedentary occupations where physical activity is typically infrequent (Smith et al., 2014). It is possible that many adults working in a sedentary occupation could have difficulty finding time to exercise each day. Because the work day takes up approximately half of all waking hours, working a full-time job can leave many people with little time to engage in physical activity during leisure time on a daily basis. Consequently, the amount of physical activity when working becomes an important component of health-related behaviors.

Sedentary adults report that they are not more active due to a variety of reasons such as exercise being too risky, exercise being too much effort, and exercise being inconvenient (Vanden Auweele et al., 1997). Owen et al. (2011) suggest that the influence of the place or environment must also be considered to understand sedentary behavior. Furthermore, an Ecological Momentary Assessment of physical activity and sedentary behavior could provide a means to better measure activity across the day (Dunton et al., 2012). Although there is considerable evidence suggesting the need for better examining levels of physical activity across the day, few studies have examined how the design characteristics of work desks may impact daily functioning.

Studies suggest that individuals who hold desk-based or computer-based jobs have higher levels of sedentary behavior (Hill and Peters, 1998; Hill et al., 2003). Work place programs have been initiated to counter this trend. Most of these work place programs are designed to increase physical activity through encouraging the worker to walk around or use stairs more during the work day. Although many work place programs may decrease sedentary behavior to some extent, they often require the worker to purposely leave the workstation, something that many workers could find difficult to do. Furthermore, work place interventions requiring that workers be active away from their workstations are fundamentally limited since they are focused on available time during the work day when employees can choose to be away from their normal sedentary workstation.

One method to reduce sedentary time is to integrate activity workstations in the work place allowing individuals to work on a desk top or a computer while being physically active. Activity workstations allow individuals to complete desk-based or computer-based tasks while moving at a low level of physical exertion usually through walking at a normal pace or by pedaling relatively slowly. Although activity workstations are a relatively new idea, they offer some useful benefits over more traditional work place interventions of encouraging employees to simply move more since the employees are not required to choose between working and being physically active. Furthermore, studies suggest that using activity workstations can increase the level of energy expenditure in the individual (Tudor-Locke et al., 2014) and may also have health benefits (Carson et al., 2014). Activity workstations may also have a positive benefit on stress and affect (Sliter and Yuan, 2015). These effects on meta-cognitive measures, such as stress and affect, are of particular interest when considering implementing workstations into settings that are normally sedentary in nature. Using workstations to provide a positive meta-cognitive effect of being physically active could encourage engaging in physical activity and, as such, increase physical activity across the lifespan.

Relatively little research has investigated the effects of using activity workstations on performance or on psychological variables (Rhodes et al., 2012). Some evidence suggests that activity workstations can result in performance decrements on tasks such as typing speed and motor skills (Straker et al., 2009; Ohlinger et al., 2011) while other studies have concluded that working on an activity workstation did not negatively affect performance (Carr et al., 2014). Furthermore, little research has examined the effect of activity workstations on meta-cognitive factors. Two studies have concluded that using activity workstations resulted in decreases in reported stress (Edelson and Danoffz, 1989; Sliter and Yuan, 2015) and beneficial effects on affect (Sliter and Yuan, 2015). Sliter and Yuan (2015) also suggested that the type of workstation (walking versus cycling) could affect psychological benefits of using the workstations.

The purpose of the current study is to examine how individuals perform while riding on a stationary bike with a desk top (FitDesk) and while seated at a normal desk (traditional desk) as well as their perceptions of working on the tasks at the two types of desks. We hypothesize that the participants will perform equally well on the FitDesk and on the traditional desk when completing complex cognitive tasks. We also hypothesize that the participants will report improved affect, motivation, morale, and engagement, when working on the FitDesk than the traditional desk.

## Materials and Methods

### Participants

The participants were 38 university students [average age: 19.64 (*SD* = 1.05), 11 males, 27 females] who were in good physical and mental health and did not report tobacco use. The experimental protocol was approved by the university Institutional Review Board and all participants signed an informed consent form before the experimental study. All participants were compensated $25 for completing the study as well as credit for completion of a research component of an introductory psychology class.

### Procedures

This study compares performance when using a FitDesk (Revo Innovations LLC, Antioch, TN, USA) to performance when sitting at a traditional desk. The FitDesk is a silent, stationary bike with a desk top which allows the user to work on a laptop or tablet while pedaling the bike. Participants were required to ride at a comfortable slow pace similar in exertion to a normal walking pace when using the FitDesk. In the current study, participants completed the tasks once while engaging in light physical activity at the FitDesk and once when seated at a traditional desk.

Prior to the onset of the experimental sessions, participants were required to complete an adaptation period using the FitDesks in the university library for two one-hour periods, while completing tasks of their choice. The participants signed in and out using a QR code and recorded the odometer reading on the FitDesk before and after their session using a provided form. After the adaptation period, the participants completed two experimental sessions at least 24 h apart and within one week of the adaptation period. Each testing session lasted approximately 45 min and took place between 8:00 AM and 7:00 PM. The participants could choose which time of the day to attend the testing sessions. Most participants (82%) chose the same time of the day for both testing sessions to fit the sessions into their course schedule.

The participants completed two cognitive tasks at their assigned desk (FitDesk or traditional desk) followed by a set of subjective surveys during each testing session. The conditions (FitDesk or traditional desk) and the two cognitive tasks were counterbalanced across the testing dates.

### Cognitive Measures

The Law School Admission Test (LSAT) includes a standardized test of verbal logical reasoning. The questions are based on information provided in a short passage. The reader is required to determine the correct answers based on the presented material. The questions evaluate the ability of the individual to analyze and critically evaluate information and arguments as well as offer insight into their ability to apply concepts or rules in a variety of situations (Law School Admission Council). The logical reasoning section of the LSAT exam has been reported to have 86% reliability (Wainer and Thissen, 1996). For the current study, participants were asked to complete as many questions as possible from a maximum of 19 questions within a 25-min period. The task was verbally described to participants at the start and participants were provided an opportunity to ask questions. Two versions of the logical reasoning test were used.

Raven’s Standard Progressive Matrices (SPM) test is often used as a test of nonverbal reasoning (Lynn et al., 2004). The SPM is a measure of an individual’s ability to extract patterns from several pictures of geometric designs and generate new ideas about complex situations (Raven, 2000). High levels of both internal consistency and test-retest reliability have been reported on the SPM (Raven et al., 2000). The current study used two sets of SPM figures each containing 18 items. The SPM test was verbally described to participants before beginning the task and participants were provided an opportunity to ask questions. The participants were asked to correctly complete as many items as possible in 10 min.

### Subjective Measures

After the cognitive tasks, the participants filled out a short set of subjective surveys. They first completed Borg’s Rating of Perceived Exertion Scale (RPE; Borg, 1982, 1990). The RPE provided a subjective measure of exercise intensity. A meta-analysis found that the RPE is related to physiological measures of physical exertion with validity coefficients ranging from 0.57 to 0.72 (Chen et al., 2002). The participants rated their level of physical exertion on a scale from 0 (nothing at all) to 10 (very, very strong).

Next the participants completed the positive and negative affect schedule (PANAS). The PANAS contains 20 affective-related words and uses a 5-point Likert scale from not at all to extremely. Watson et al. (1988) report that the PANAS has high internal reliability for the positive (Chronbach’s α of 0.86–0.90) and negative (Chronbach’s α of 0.84–0.87) scales.

The participants then filled out a single item question on motivation followed by the five-item Morale Scale evaluating energy, drive, enthusiasm, eagerness, and morale while completing the testing session (Britt et al., 2013). The questions were assessed on a 5-point Likert scale from very low to very high. The Morale Scale has been shown to have a Cronbach’s α of 0.93 (Britt et al., 2013).

Last, participants completed the Engagement Scale using a 5-point Likert scale from strongly disagree to strongly agree (Britt et al., 2010). Participants responded to six items assessing subjective performance, absorption, and attention. The Engagement Scale has been shown to be a predictor of performance in academic settings (Britt et al., 2010).

### Statistical Analysis

Individual responses that were greater than three standard deviations away from the mean for the item were removed from data analysis. This occurred only with the RPE where three subjects’ were removed from the analysis. The LSAT and SPM were scored as percent correct. The PANAS was scored based on established metrics to create a positive and negative score (Watson et al., 1988). The responses on the Morale and Engagement Scales were averaged for each scale (Britt et al., 2010, 2013).

SPSS 22 (SPSS Inc., Chicago, IL, USA) was used for all data analyses. A repeated-measures MANOVA was used to determine if there were differences between the FitDesk and traditional desk conditions.

## Results

The descriptive results for each variable (means, standard deviations, and 95% confidence intervals) are shown in **Table 1**. There were no significant differences in performance on the LSAT or the SPM when comparing performance on the FitDesk to performance at the traditional desk.

**Table 1:** Descriptive statistics: means, standard deviations, and 95% confidence intervals.

Variable	*M*	*SD*	95% CI lower	95% CI upper
LSAT
FitDesk	51.52	17.91		
Traditional Desk	49.03	18.88	-2.67	7.66
Raven’s SPM
FitDesk	79.53	15.72		
Traditional Desk	84.65	11.07	-10.64	0.41
Rating of perceived exertion
FitDesk	2.01	1.08		
Traditional desk	0.48**	0.99	1.18	1.88
Positive PANAS
FitDesk	28.26	5.84		
Traditional desk	23.47**	5.81	2.69	6.89
Negative PANAS
FitDesk	11.71	1.58		
Traditional desk	12.18	3.17	-1.53	0.58
Motivation
FitDesk	3.26	0.64		
Traditional desk	2.87*	0.81	0.12	0.67
Morale
FitDesk	3.16	0.62		
Traditional desk	2.65**	0.68	0.25	0.78
Engagement
FitDesk	3.75	0.53		
Traditional desk	3.61	0.58	-0.06	0.33

*p = 0.005; **p < 0.001.

Participants reported greater physical exertion when using the FitDesk [*F*(1, 34) = 78.386, *p* < 0.001, and $\eta_{p}^{2}$ = 0.697]. Participants reported significantly greater positive affect on the PANAS [*F*(1,34) = 19.542, *p* < 0.001, and $\eta_{p}^{2}$ = 0.365] when using the FitDesk but did not report a difference on the negative affect. Participants also reported higher levels of motivation [*F*(1,34) = 5.79, *p* = 0.009, and $\eta_{p}^{2}$ = 0.187] and higher levels of morale [*F*(1,34) = 12.010, *p* = 0.001, and $\eta_{p}^{2}$ = 0.261] when working on the FitDesk. In contrast, there was no difference in engagement between the conditions.

## Discussion

The current results suggest that the use of the FitDesk positively affects subjective states while not decreasing complex cognitive performance as measured by the LSAT logical reasoning and the SPM. More specifically, using the FitDesk resulted in increased levels of positive affect, motivation, and morale. In addition, when using the FitDesks, participants reported greater physical exertion than when using the traditional desk. However, the average subjective exertion level for both workstations remained quite low (FitDesk = weak; traditional desk = very, very weak).

The current results supported our first hypothesis that working on the FitDesk would not negatively affect complex cognitive performance. The lack of significant effects on logical reasoning and fluid intelligence tasks are consistent with previous findings that activity workstations do not impact cognitive functioning (Ohlinger et al., 2011). This suggests that activity workstations could be used in the work place and educational settings without fear of negatively affecting complex cognitive performance. This finding could have a profound effect in work places and other environment settings where people are required to sit for long periods of time (e.g., waiting rooms, airports). As suggested by Proper et al. (2011), interventions to reduce sedentary behavior are needed in many environments in modern society. Furthermore, attempting to separate work from other aspects of life may not be a meaningful way to evaluate health-related behaviors or long-term health risks (Panelli and Gallagher, 2003). Instead it is important to consider physical activity throughout the day and provide recommendations that can be implemented in modern societies that are increasingly requiring sedentary work. Because sedentary behavior has become common practice in many work places, implementing activity workstations could decrease sedentary activity while allowing workers to complete their responsibilities.

The current results partially supported our second hypothesis that working while on the FitDesk would improve affect, motivation, morale, and engagement. We found a significant improvement in positive affect, motivation, and morale but not in engagement as measured by Britt et al. (2010) engagement scale. Improvements in positive mood states and morale are seen following exercise (Tate and Petruzzello, 1995; Reed and Ones, 2006; Liao et al., 2015) suggesting that light activity when using workstations may have similar effects as moderate to vigorous exercise. The current results indicate that light physical activity on a cycling workstation while completing cognitive tasks can improve positive affect and morale. These findings contrast with a study concluding that cycling workstations resulted in reduced satisfaction (Sliter and Yuan, 2015). However, the cycling workstations used in the Sliter and Yuan study were not FitDesks and instead were separate cycle units set up near a desk top which seemed to bring about participant discomfort. In contrast, FitDesks are ergonomically designed to counter this possible effect with the desk top located at a comfortable distance from an adjustable height seat.

It is important to note that the use of the FitDesks in the current study had a positive impact on the emotions and feelings of the participants. The participants were more positive, had greater motivation, and better morale when completing required complex cognitive tasks while being active and working on the FitDesk than while being inactive and working at a traditional desk. This suggests that individuals could enjoy using activity workstations, such as the FitDesk, at work or in educational settings where environments currently encourage sedentary activity. This positive feeling from individuals could help increase the amount of physical activity that individuals choose to participate in daily. In addition, because the light physical activity can be completed while doing tasks, this makes more time available for physical activity during the day when many individuals say that they do not have time for a stand-alone exercise period. Additional research is required using activity workstations to better document how well activity workstations can be integrated into different sedentary environments. More research is also needed on additional meta-cognitive factors such as acceptance of using activity workstations and the impact of completing tasks on activity workstations as well as potential mediating variables.

The improvement in positive affect seen in the current study could have important work place implications. Positive affect is associated with improved problem solving and decision making which can lead to flexible and creative cognitive processing (Isen, 2001) and can help facilitate coping mechanisms and healthy behaviors in individuals (Aspinwall, 1997). Improved positive affect and motivation are also related to responsible work behavior (Isen and Reeve, 2005) while increased motivation is related to persistence (Glastra et al., 2004). The results from the current study suggest that light physical activity could produce positive mental states while completing necessary tasks which in turn could improve over-all morale in a variety of work settings.

It is interesting to note there was no significant change in negative affect on the PANAS while there was an improvement in positive PANAS when using the FitDesk. Previous studies have shown that negative affect is not impacted by age (Mroczek and Kolarz, 1998) or by sleep deprivation (Pilcher et al., 2015). Furthermore, research suggests that negative affect does not change in persons focused on goal-driven behaviors (Mogg and Bradley, 1998). Previous research also indicates that steady, lower levels of negative affect are related to an increase in life satisfaction (Pilcher, 1998). These results suggest that negative affect may be a more stable phenomenon than positive affect. As such, it seems unlikely that negative affect would be easily altered by light physical activity.

The current study has some limitations. Participants were not screened for physical fitness levels prior to the start of the study. However, the participants were healthy and young and they reported a low level of physical exertion ranging from weak to very, very weak. Because of the low level of activity, the physical fitness level is less of a concern in the present study. Future studies can be designed to include a measure of physical fitness in research examining light physical activity to address this issue. In addition, the current study used college students as participants which could make it difficult to generalize the findings to older adults. However, student and nonstudent samples tend to agree about 80% of the time (Highhouse and Gillespie, 2008) suggesting that the negative effects of student sampling is limited.

## Conclusions

The present study is among the first research studies examining performance and meta-cognitive outcomes when using an activity workstation versus a traditional desk. The results indicate that light physical activity on a stationary bicycle had no detrimental effect on performance on complex cognitive tasks but did result in an improvement in meta-cognitions related to daily functioning, affect, and decision making. Furthermore the improvement in affect, motivation, and morale, suggests that light activity when working on an activity workstation may help encourage light physical activity when completing necessary tasks as well as desired activities (e.g., TV and video games). It is also possible that a positive feeling associated with physical activity could encourage individuals to be more physically active, thus increasing physical activity across the lifespan. Together these findings support the potential of implementing activity workstations in the workplace and in educational settings. Additional research is needed examining the effects of activity workstations in different settings and with different populations. However, the current results suggest that making activity workstations more available could help decrease the amount of sedentary behavior experienced by many adults with little disruption to their daily work. Implementing activity workstations in settings where individuals are expected to sit (e.g., the workplace, educational settings, waiting rooms, and airports) could decrease sedentary activity without negatively affecting performance and could have positive effects on affect, motivation, and morale.

## Author Contributions

JP conceived of the study with input from VB. JP provided oversight for VB in developing the methods for the study. VB carried out the data collection and initial data analysis under direction by JP. VB wrote an early draft of the manuscript. JP revised and completed the writing of the manuscript. All authors read and approved the final manuscript.

## Conflict of Interest Statement

The authors declare that the research was conducted in the absence of any commercial or financial relationships that could be construed as a potential conflict of interest.
